# Forecasting the Incidence and Prevalence of Patients with End-Stage Renal Disease in Malaysia up to the Year 2040

**DOI:** 10.1155/2017/2735296

**Published:** 2017-02-28

**Authors:** Mohamad Adam Bujang, Tassha Hilda Adnan, Nadiah Hanis Hashim, Kirubashni Mohan, Ang Kim Liong, Ghazali Ahmad, Goh Bak Leong, Sunita Bavanandan, Jamaiyah Haniff

**Affiliations:** ^1^National Clinical Research Centre, Kuala Lumpur, Malaysia; ^2^Clinical Research Centre, Serdang Hospital, Kajang, Malaysia; ^3^Department of Nephrology, Kuala Lumpur Hospital, Kuala Lumpur, Malaysia; ^4^Department of Nephrology, Serdang Hospital, Kajang, Malaysia; ^5^Malaysian Health Performance Unit, Ministry of Health, Kuala Lumpur, Malaysia

## Abstract

*Background*. The incidence of patients with end-stage renal disease (ESRD) requiring dialysis has been growing rapidly in Malaysia from 18 per million population (pmp) in 1993 to 231 pmp in 2013.* Objective*. To forecast the incidence and prevalence of ESRD patients who will require dialysis treatment in Malaysia until 2040.* Methodology*. Univariate forecasting models using the number of new and current dialysis patients, by the Malaysian Dialysis and Transplant Registry from 1993 to 2013 were used. Four forecasting models were evaluated, and the model with the smallest error was selected for the prediction.* Result*. ARIMA (0, 2, 1) modeling with the lowest error was selected to predict both the incidence (RMSE = 135.50, MAPE = 2.85, and MAE = 87.71) and the prevalence (RMSE = 158.79, MAPE = 1.29, and MAE = 117.21) of dialysis patients. The estimated incidences of new dialysis patients in 2020 and 2040 are 10,208 and 19,418 cases, respectively, while the estimated prevalence is 51,269 and 106,249 cases.* Conclusion*. The growth of ESRD patients on dialysis in Malaysia can be expected to continue at an alarming rate. Effective steps to address and curb further increase in new patients requiring dialysis are urgently needed, in order to mitigate the expected financial and health catastrophes associated with the projected increase of such patients.

## 1. Introduction

End-stage renal disease (ESRD), now termed chronic kidney disease (CKD) stage five is a state of permanent loss of renal function when measured or calculated glomerular filtration rate is less than 15 ml/min permanently. Worldwide, the number of ESRD patients is growing rapidly in developed and developing countries, fueled by aging populations and a pandemic of chronic noncommunicable diseases especially diabetes mellitus and hypertension. Current projections indicate that, by 2030, the global population of ESRD patients living on dialysis may exceed 2 million [1].

In Malaysia, the incidence and prevalence of patients with ESRD has been on an upward trend for the past 20 years. The increase in the dialysis population is attributable to the increasing availability of haemodialysis treatment facilities and easier access to public or subsidised funding, especially in the nongovernmental sector. The 22nd Report of the Malaysian Dialysis and Transplant Registry in 2013 recorded a total of 32,026 patients receiving dialysis treatment, 29,192 on haemodialysis (91%) and 2,834 on peritoneal dialysis (9%) [2]. In 1993, 358 new ESRD patients were treated with dialysis, 2,629 in 2003, while in 2013 the number steeply increased to 6,985. The incidence of patients with ESRD on dialysis was 18 per million population (pmp) in 1993. Subsequently, the numbers doubled from  104 pmp in 2003 to 231 pmp in 2013. The prevalence of patients with ESRD on dialysis was 71 pmp in 1993, rising subsequently more than twofold from 415 pmp in 2003 to 1,059 pmp in 2013 [2].

As the risk of ESRD increases with age, the aging population in Malaysia is expected to have a large impact on the number of incident dialysis patients. A study by Wakasugi et al. observed the trend in the incidence rates of dialysis in Japanese population between 2008 and 2012. The total number of incident dialysis patients was predicted to increase by 12.8% from 36,590 in 2012 to 41,270 in 2025, with higher increment in the oldest age group (≥85 years). Male and female patients were expected to increase by 92.6% and 62.2%, respectively [3].

Looking at the trend in the past 10 years, there is a need to do forecasting studies of ESRD patients to create awareness among the policy makers and the public with regard to the magnitude of the disease. Accurate prediction can be used to facilitate the policy making, decisions on optimal health care allocation and appropriate financial planning and allow for more appropriate use of health care resources for the Malaysian population. This study aims to use time-series techniques on the available historical data to find the best model with the smallest error that can forecast the number of new ESRD patients in Malaysia in the future. Hence, the purpose of this study is to describe the trend of incidence and prevalence of ESRD patients in Malaysia from 1993 to 2013. The study also aims to determine the best univariate forecasting model which produces the smallest root mean squared error (RMSE), mean absolute percentage error (MAPE), and mean absolute error (MAE), subsequently to predict the incidence and prevalence of persons with ESRD up until the year 2040.

## 2. Methodology

This study was registered and approved by the ethics committee of the National Medical Research Register (NMRR-13-1783-18577). Univariate forecasting modeling was constructed using the new patients (incidence) with a confirmed diagnosis of ESRD on renal replacement therapy (RRT) in Malaysia by year, from 1993 to 2013. The univariate forecasting models were applied since these techniques also generate good forecasts especially when the trend is stable [4]. Four types of time-series analyses methods were assessed, namely, the naive with trend method, the double exponential smoothing method, Holt's method, and autoregressive integrated moving average (ARIMA) modeling method. Forecasting performance is evaluated by estimating the RMSE, MAPE, and MAE and the model with the smallest forecast error was selected to be used in predicting the incidence and prevalence of ESRD up until 2040.

The incidence and prevalence rates of ESRD patients were calculated using population estimates from the Department of Statistics, Malaysia. Meanwhile, based on a previous local study in 2001, the cost of dialysis treatment for each patient was estimated to be RM 30,000 (USD 7,500) per patient, per year [5]. The cost per patient was estimated to be within RM 29,092 to RM 33,642 [5]. The estimated future costs did not incorporate the expected changes due to various factors like changes in foreign exchange rate, consumer price index, changes in the cost of utilities, emoluments, consumables, hardware, pharmaceuticals, and others. The analysis was conducted using Microsoft Excel and R software (R Development Core Team (2013). R: A language and environment for statistical computing. R Foundation for Statistical Computing, Vienna, Austria. ISBN 3-900051-07-0, URL https://www.R-project.org.).

## 3. Result

The yearly incidence and prevalence of dialysis patients (haemodialysis and peritoneal dialysis) in Malaysia have been on the increasing trend for the past 20 years. Based on the four types of univariate models measured, the ARIMA modeling which gives the smallest RMSE, MAPE and MAE was selected to be used for predicting the number of new dialysis patients from 2014 to 2040. ARIMA (0, 2, 1) was used for both projection of incidence of dialysis patients (RMSE = 135.50, MAPE = 2.85, and MAE = 87.71) and also prevalence of dialysis patients (RMSE = 158.79, MAPE = 1.29, and MAE = 117.21) (Table 1).

**Figure 1:**
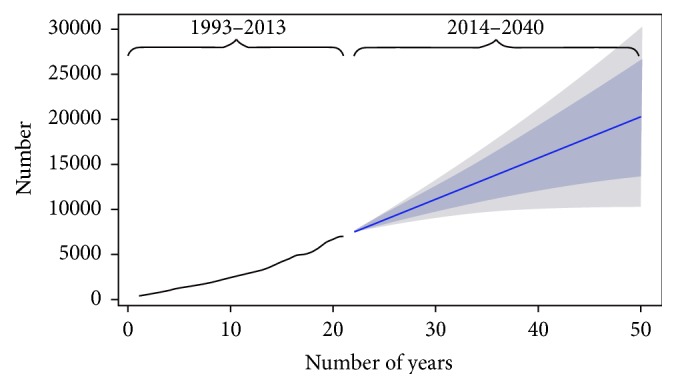
Trend and forecast values for incidence of ESRD patients (ARIMA [0, 2, 1]).

**Figure 2:**
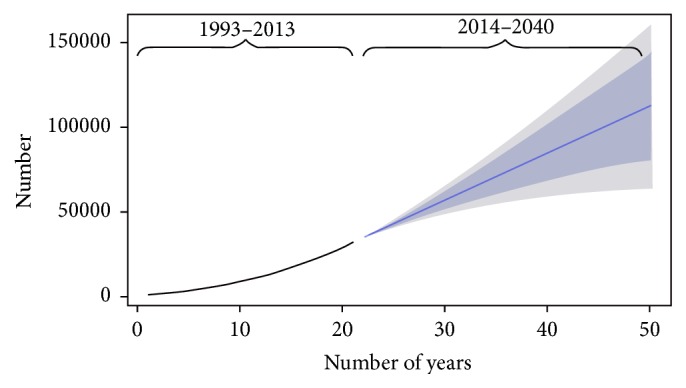
Trend and forecast values for prevalence of ESRD patients (ARIMA [0, 2, 1]).

Based on the model selected, the projected number of incidence and prevalence dialysis patients in Malaysia will increase as shown in Figures 1 and 2. The estimated incidence of new dialysis patients in Malaysia in 2020 is 10,208 cases and 19,418 in 2040. Meanwhile, the estimated prevalence is 51,269 and 106,249 cases in 2020 and 2040, respectively. With such projected prevalence, the estimated costs for the treatment are USD 384,517,500 and USD 796,867,500 in the years 2020 and 2040, respectively (Table 2).

## 4. Discussion

Based on projections made by the Department of Statistics (DOS), Malaysia is expected to reach aging population status by the year 2035, at which point 15 per cent of the total population will be 60 years and older [6]. As the number of older adults is projected to increase dramatically over the next twenty years, this will pose major challenges to our health care systems, as there will be greater healthcare utilization for comorbid conditions among this population [7].

With the rise in conditions such as obesity, diabetes, and hypertension in the middle age population, it is likely that the future prevalence of chronic kidney disease (CKD) will increase further among the elderly. It is a significant concern with both an increasing incidence of treated kidney failure with dialysis and a high prevalence of earlier stages of CKD. CKD is silent and irreversible. If CKD is not detected early or managed properly, the condition will progress to ESRD which will result in the need for renal replacement therapy with either dialysis or kidney transplant. Additionally, ESRD also can lead to mental illnesses such as anxiety and depression which will affect the patients' quality of life [8].

Renal replacement therapy (RRT) in Malaysia has shown an exponential growth since 1990 [9]. The rate of incidence of end-stage renal disease requiring RRT is forecasted to be 3.02 (per 10,000 population) by the year 2020 and to increase further to 3.89 in 2030 and reaching a rate of 4.68 in 2040. There is a projected 1.5-fold increase in overall number of patients on dialysis from 6,985 in 2013 to 10,208 in 2020 and a further 1.5-fold increase to 14,813 in 2030, with a 1.3-fold increase to 19,418 in 2040. This is a serious health and economic burden, as patients with ESRD consume a vastly disproportionate amount of financial and human resources.

The cost of CKD to the National Health System in the United Kingdom from the year 2009 to 2010 is estimated to be £1.44 to £1.45 billion, which is about 1.3% of all NHS expenditure in that year. More than half of this amount was spent on RRT, which was provided for 2% of the CKD population [10]. In Malaysia, we projected the costs based on costs estimated by Hooi and colleagues [5]. They estimated the costs were within RM 29,092 to RM 33,642 per patient dialysed at Ministry of Health facilities. By taking average cost of RM 30,000 per patient, the estimated cost incurred to treat all patients with dialysis in the year 2040 is USD 796,867,500 (including new patients at USD 145,635,000) which is more than 10 times compared to the amount in the year 2000. Since the cost was estimated based on year 2001, we postulate that the future cost is higher than what we have estimated due to various factors such as changes in the currency exchange rate, consumer price index, and possibility of the increased costs of the current treatment. Hence, the future costs that is estimated by this study can only become a rough projection to indicate the magnitude and burden of disease.

Careful projections of the demand for dialysis services are important to assist healthcare planners in forecasting the need for equipment, facilities, and personnel [11]. Even so, the setting up of more dialysis centres is not the way to go, as it cannot keep up with the rising need for RRT. More effective prevention, intervention, and early detection programs for renal diseases should be put into place to increase awareness among the public and medical personnel alike.

The health care system should place an emphasis on education and on creating awareness on CKD among the general public. They should be aware of the comorbid conditions that lead to CKD and should learn to adapt lifestyle changes to manage their health properly. Medical professionals should be trained adequately and expanded surveillance and screening systems for chronic diseases made available and easily accessible. The Disease Control Priorities Project offers recommendations on these measures, supporting the establishment of international centers of excellence for CKD that can work in tandem with centers of cardiovascular diseases (CVD) and diabetes [12]. Also, there are collaborations between the International Society of Nephrology and Kidney Disease Improving Global Outcomes with societies such as the International Diabetes Federation (IDF), WHO, and the World Bank to influence global and national health decision-making with regard to the growing burden of CKD and chronic diseases [13, 14].

On a larger scale, there needs to be better alignment of disease funding with burden of disease, transparency of projects, and data availability and better sharing of knowledge with more concerted activities to address the problem of chronic kidney disease (CKD) [15]. More specific cost-effective strategies need to be implemented to cope with the rising demand for RRT. Specific interventions may be used, for example, the increased use of angiotensin-converting enzyme inhibitors and angiotensin receptor type 2 antagonists to slow the rate of decline in renal function [16]. In diabetics, glycaemic control may be improved with the use of newer antidiabetics agents, thus lowering the rate of renal loss. Furthermore, antismoking initiatives may also help slow the progression of chronic kidney disease to ESRD [17].

With these efforts in place, we can perhaps better respond to the challenging burden of CKD and the rising need for RRT in the country. As for conclusions, the predicted number of incidence and prevalence of dialysis patients in Malaysia seem to be increasing over the years until 2040. The number of patients commencing dialysis in Malaysia is predicted to be more than tripled, from 32,026 in 2013 to 106,249 in 2040 (including new patients from 6,985 in 2013 to 19,418 in 2040). The pmp of new dialysis patients in Malaysia is estimated to increase by almost twofold; from 231 pmp in 2013 to 467 pmp in 2040. In view of these trends especially with the trend of aging population in the country [7], there is a need for more targeted, unified, and coordinated efforts to better manage the issues that may arise from this pattern.

The limitation of study is that the univariate forecasting model was used. Therefore, this model did not incorporate other important factors such as age, prevalence of comorbidities (i.e., diabetes mellitus and hypertension), and lifestyle factors. Most of these data are difficult to be retrieved; hence, applying a univariate forecasting model especially for stable time-series pattern is necessary. The forecasted incidence is assumed to be true based on historical data when there is no change in the populations' behavior and no specific interventions have been made. However, with public awareness and lifestyle changes with effective changes in healthcare management, it is possible that in the future the true incidence and prevalence will be lower than what has been estimated.

## Figures and Tables

**Table 1:** Comparison of forecast error from number of ESRD patients, 1993–2013.

Method	Incidence				Prevalence			
	Parameter	RMSE	MAPE	MAE	Parameter	RMSE	MAPE	MAE
Naive with trend	—	165.12	3.73	116.37	—	195.81	1.81	160.16
Double exponential smoothing	*α* = 0.6577	151.33	5.64	110.87	*α* = 0.9999	205.96	2.72	169.47
Holt	*α* = 0.9999, *β* = 0.3221	135.40	3.24	90.26	*α* = 0.9999, *β* = 0.9999	199.78	3.09	167.14
ARIMA (0, 2, 1)	—	135.50	2.85	87.71	—	158.79	1.29	117.21

**Table 2:** Estimated cost to treat patients with ESRD in year 2000 to 2040.

Year	Est. pop. (in '000)	Incidence				Prevalence			
		Number	Rate (per 10,000 pop.)	Est. cost (RM mil)	Est. cost (USD mil)	Number	Rate (per 10,000 pop.)	Est. cost (RM mil)	Est. cost (USD mil)
2000	23,494.90	1,853	0.79	55.59	13.90	6,702	2.85	201.06	50.27
2005	26,045.50	3,167	1.22	95.01	23.75	13,356	5.13	400.68	100.17
2010	28,588.60	5,305	1.86	159.15	39.79	23,709	8.29	711.27	177.82
2015^a^	31,186.10	7,906	2.54	237.18	59.30	37,524	12.03	1,125.72	281.43
2020^a^	33,782.40	10,208	3.02	306.24	76.56	51,269	15.18	1,538.07	384.52
2025^a^	36,022.70	12,511	3.47	375.33	93.83	65,014	18.05	1,950.42	487.61
2030^a^	38,062.20	14,813	3.89	444.39	111.10	78,759	20.69	2,362.77	590.69
2035^a^	39,879.30	17,116	4.29	513.48	128.37	92,504	23.20	2,775.12	693.78
2040^a^	41,503.10	19,418	4.68	582.54	145.64	106,249	25.60	3,187.47	796.87

Est. = estimated; pop. = population.

a estimates number of cases from the selected model (ARIMA [0, 2, 1]).

Each ESRD patient is estimated to incur cost for RM 30,000 (on estimated rate of 1.00 USD = 4.00 MYR).

Source of estimated population from Department of Statistics, Malaysia.
